# Elimination of Non-cytopathic Bovine Viral Diarrhea Virus From the LFBK-α_v_β_6_ Cell Line

**DOI:** 10.3389/fvets.2021.715120

**Published:** 2021-08-11

**Authors:** Ashley R. Gray, Britta A. Wood, Elisabeth Henry, Donald P. King, Valérie Mioulet

**Affiliations:** World Reference Laboratory for Foot-and-Mouth Disease, The Pirbright Institute, Woking, United Kingdom

**Keywords:** bovine viral diarrhea virus, LFBK-αvβ6, DB772, WRL-LFBK-αvβ6, vesicular disease virus susceptibility, foot-and-mouth disease virus

## Abstract

The LFBK-α_v_β_6_ cell line is highly sensitive for the isolation of foot-and-mouth disease virus (FMDV) and porcinophilic vesicular viruses. However, LFBK-α_v_β_6_ cells are contaminated with a non-cytopathic bovine viral diarrhea virus (BVDV), which complicates handling procedures in areas where other cell lines are maintained, as well downstream use of viral isolates. In this study, we used an aromatic cationic compound (DB772) to treat LFBK-α_v_β_6_ cells using an approach that has been previously used to eliminate persistent BVDV from fetal fibroblast cell lines. After three cell passages with 4 μM DB772, BVDV could no longer be detected in unclarified cell suspensions using a pan-pestivirus real-time RT-PCR assay, and remained undetectable after treatment was stopped (nine passages) for an additional 28 passages. The analytical sensitivity of the DB772-treated LFBK-α_v_β_6_ cultures (renamed WRL-LFBK-α_v_β_6_) to titrations of FMDV and other vesicular virus isolates was comparable to untreated LFBK-α_v_β_6_ cells. These new BVDV-free cells can be handled without the risk of cross-contaminating other cells lines or reagents, and used for routine diagnostics, *in vivo* studies and/or preparation of new vaccine strains.

## Introduction

LFBK-α_V_β_6_ cells are a porcine kidney cell line that was transduced to express the bovine α_V_β_6_ integrin receptor in order to provide a highly sensitive, continuous cell line for the propagation of foot-and-mouth disease virus (FMDV) [(1); correction (2)]. LaRocco et al. (1) demonstrated that over-expression of α_V_β_6_ integrin, a known cellular receptor for FMDV, enhanced susceptibility to a range of FMDVs relative to other continuous cell lines (i.e., BHK, IB-RS-2, MVPK, LFBK, and LK), whilst maintaining sensitivity to vesicular viruses that are clinically indistinguishable from FMD. Subsequently, Fukai et al. (3) demonstrated that LFBK-α_V_β_6_ cells have similar susceptibility to non-epithelium FMDV clinical samples as ZZ-R 127 cells.

We previously validated the use of LFBK-α_V_β_6_ cells for diagnostic purposes within the World Reference Laboratory for FMD (WRLFMD) (4). Our results demonstrated that LFBK-α_V_β_6_ cells had similar analytical sensitivity to FMDV epithelium suspensions as primary bovine thyroid cells (BTY), which had previously been identified as the most sensitive cell system for FMDV isolation (5). Additionally, the LFBK-α_V_β_6_ cells had enhanced susceptibility to porcine-adapted FMDVs compared to IB-RS-2 cells (4). Unfortunately, the LFBK-α_V_β_6_ cell line is persistently infected with a non-cytopathic bovine diarrhea virus (BVDV; family *Flaviviridae*, genus *Pestivirus*) (Rodriguez, personal communication), which complicates the use of these cells due to concerns about cross-contamination of other cell lines and downstream applications, including vaccine production and preparation of challenge viruses for *in vivo* studies.

The presence of a non-cytopathic BVDV in cells is not a novel occurrence. Multiple studies have documented non-cytopathic BVDV strains present in fetal bovine serum (6–10), which likely lead to the subsequent contamination of numerous cell lines, including many non-bovine cultures (11–13). Given the negative impact of BVDV in the cattle industry and as a laboratory contaminant, numerous studies have evaluated chemical compounds on their ability to inactivate or inhibit BVDV [reviewed in (14)]. One aromatic cationic compound, DB772, has been shown to prevent and eliminate non-cytopathic BVDV from persistently infected fetal fibroblast cells without causing cytotoxicity (15, 16), as well as having antiviral properties *in vivo* (17).

The aim of this study was to eradicate BVDV from LFBK-α_V_β_6_ cells using DB772, following similar procedures to those described in Givens et al. (16). Unclarified cell suspensions collected during and after DB772 treatment were tested for the presence of BVDV genome using a pestivirus real-time RT-PCR assay (rRT-PCR). Cell line sensitivity after treatment with DB772 was assessed by performing comparative titrations with a range of vesicular viruses alongside the original LFBK-α_v_β_6_ cell line.

## Materials and Methods

All experiments were conducted within high-containment laboratories at The Pirbright Institute that meet the *Minimum Biorisk Management Standards for Laboratories Working with Foot-and-Mouth Disease Virus* of the European Commission for the Control of Foot-and-Mouth Disease (18).

### Antiviral Compound (DB772)

The compound 2-{5-[4-(4,5-Dihydro-1*H*-imidazol-2-yl)phenyl]furan-2-yl}-1*H*-benzo[d]imidazole 4-toluenesulfonate salt (DB772) was synthesized by NewChem Technologies (Durham, UK). A stock solution of 10 mM was prepared in dimethyl sulfoxide (Sigma) and stored at room temperature in the dark until use.

### Cells and Treatment With DB772

The LFBK-α_v_β_6_ cell line [(1), correction (2)], supplied by the Plum Island Animal Disease Center (New York, USA), was maintained as previously described (4). Briefly, LFBK-α_v_β_6_ cells (passage 19) were grown in a 175 cm^2^ cell filter-cap tissue culture flask (Cellstar, Greiner Bio-One) at 37°C in the presence of 5% CO_2_ until the monolayer reached 90–100% confluency. The cell monolayer was washed with 15 mL sterile phosphate buffer saline (PBS; Severn Biotech), followed by 15 mL 0.25% trypsin-EDTA (Gibco). After removing the trypsin, the flask was incubated at 37°C until the cells dissociated from the flask surface. The cells were then resuspended in 25 mL Dulbecco's modified Eagle medium (DMEM; Gibco) supplemented with 10% bovine serum (BS; certified BVDV negative, Gibco) (referred to DMEM + BS). Two 50 mL Falcon tubes each received 1 mL of the cell suspension and were centrifuged at 340 g for 5 min at 4°C. The pelleted cells were resuspended in 5 mL of DMEM + BS (untreated control) or 4 μM DB772 DMEM + BS, transferred to 25 cm^2^ filter-cap tissue culture flasks (TPP Techno Plastic Products) and incubated at 37°C in the presence of 5% CO_2_. After 24 h, the media was removed from the flasks and replaced with the corresponding 5 mL DMEM + BS with or without DB772. The cells were incubated at 37°C in the presence of 5% CO_2_ until the monolayers reached 90–100% confluency.

Subsequent passages were performed following the procedure above, including using 1 mL of cell suspensions (split ratio of 1:4), but with the other volumes adjusted for the use of 25 cm^2^ flasks (i.e., 3 mL sterile PBS, 3 mL 0.25% trypsin-EDTA, resuspension of cells in 4 mL DMEM + BS, and 5 mL DMEM + BS with or without DB772). At each passage, 125 μL of the cell suspension was added to 325 μL of MagMAX lysis buffer (ratio 5:13; Applied Biosystems, Thermo Fisher Scientific) and stored at −80°C until testing. Treatment with DB772 continued for 9 passages (passages 20–28), after which the cells were maintained in DMEM + BS, identical to the untreated cells. To differentiate the two cultures, the LFBK-α_v_β_6_ cells treated with 4 μM DB772 were renamed WRL-LFBK-α_v_β_6_.

LFBK-α_v_β_6_ and WRL-LFBK-α_v_β_6_ cultures were both maintained for 10 subsequent passages (passages 29–38), and at each passage, cell suspension was added to lysis buffer and stored at −80°C until testing. The WRL-LFBK-α_v_β_6_ culture was maintained for an additional 18 passages (passages 39–56), and at passages 40, 44, 48, 52, and 56 cell suspension was added to lysis buffer and stored at −80°C until testing. WRL-LFBK-α_v_β_6_ cell stocks were stored in liquid nitrogen at passages 33, 34, and 40.

### BVDV Detection

RNA was purified from unclarified cell suspensions using the MagMAX-96 viral RNA isolation kit (Applied Biosystems, Thermo Fisher Scientific) on a Kingfisher Flex extraction robot (Thermo Fisher Scientific) using an automated protocol. All extractions were performed in triplicate, including a BVDV genome positive and a negative control per extraction plate. The positive control was prepared from LFBK-α_v_β_6_ cells and generated a value of ~23 C_T_, and the negative control was a negative pig epithelium suspension (i.e., control regularly used with routine vesicular disease diagnostic testing and which contains porcine genomic material).

Purified RNA was tested by rRT-PCR using the EXPRESS One-Step Superscript qRT-PCR kit (Invitrogen, Thermo Fisher Scientific). Each well of the rRT-PCR consisted of 5 μL of RNA template and 15 μL of master mix [10 μL of EXPRESS mix, 1 μL each of forward and reverse primer (20 μM), 0.5 μL of probe (15 μM), 0.5 μL ROX reference dye (diluted 1-in-10 with nuclease free water) and 2 μL Taq polymerase]. Primers and probes targeting the conserved 5′ non translated region of the pestivirus genome (19) were used, as recommended by the OIE Manual (20) for the detection of BVDV. The following cycling conditions were used on a 7,500 Fast Real-time PCR instrument using the fast setting (Applied Biosystems, Thermo Fisher Scientific): 50°C for 15 min, 95°C for 20 s, then 50 cycles of 95°C for 3 s and 60°C for 30 s. Samples with C_T_ a value ≤40 were considered positive (20).

### Virus Titrations

Representative vesicular disease viruses were selected for testing from the WRLFMD collection. FMDV/O/KUW/4/2016 original suspension was prepared as described in Gray et al. (4) from vesicular epithelium, and is used to routinely monitor primary and continuous cell line batch-to-batch variation. In addition, the following viruses were selected for testing: swine vesicular disease virus (SVDV) UKG/77/1980 RS1, vesicular stomatitis virus (VSV) IND-1 BHK5, vesicular exanthema of swine virus (VESV) D53 PK5 RS3, and Seneca Valley virus (SVV) MN-88-36695 SK? RS3. Virus titrations were performed in parallel to compare the relative cell line sensitivities.

WRL-LFBK-α_v_β_6_ and LFBK-α_v_β_6_ cells were expanded (starting at passage 40) for the titrations; cells were seeded in Nunc flat-sided cell culture tubes (5.5 cm^2^; Thermo Fisher Scientific) and reached 90–100% confluency in 24 h with stationary incubation at 37°C. Virus stocks were serially diluted 10-fold in M25 buffer [35 mM disodium hydrogen phosphate (Sigma) and 5.7 mM potassium dihydrogen phosphate (Fisher Scientific) in sterile water]. Cells (*n* = 5 tubes per cell line per dilution/control) were washed with 2 mL sterile PBS before adding 2 mL of minimal essential media (MEM; Gibco) supplemented with 6 mL/L field antibiotics and 2% BS (Gibco). The cell tubes were then inoculated with 0.2 mL of the appropriate virus dilution and incubated with rotation at 37°C. A final read of the cells was performed after 72 h by examining under a microscope for the presence of cytopathic effect (CPE). For each cell line, viral titers were calculated using the Spearman-Karber method and expressed as Log_10_ TCID_50_/mL. Three independent titrations were performed per virus and average viral titers were compared between cell lines using *t*-tests (GraphPad Prism 9.1.0).

## Results

**Figure 1:**
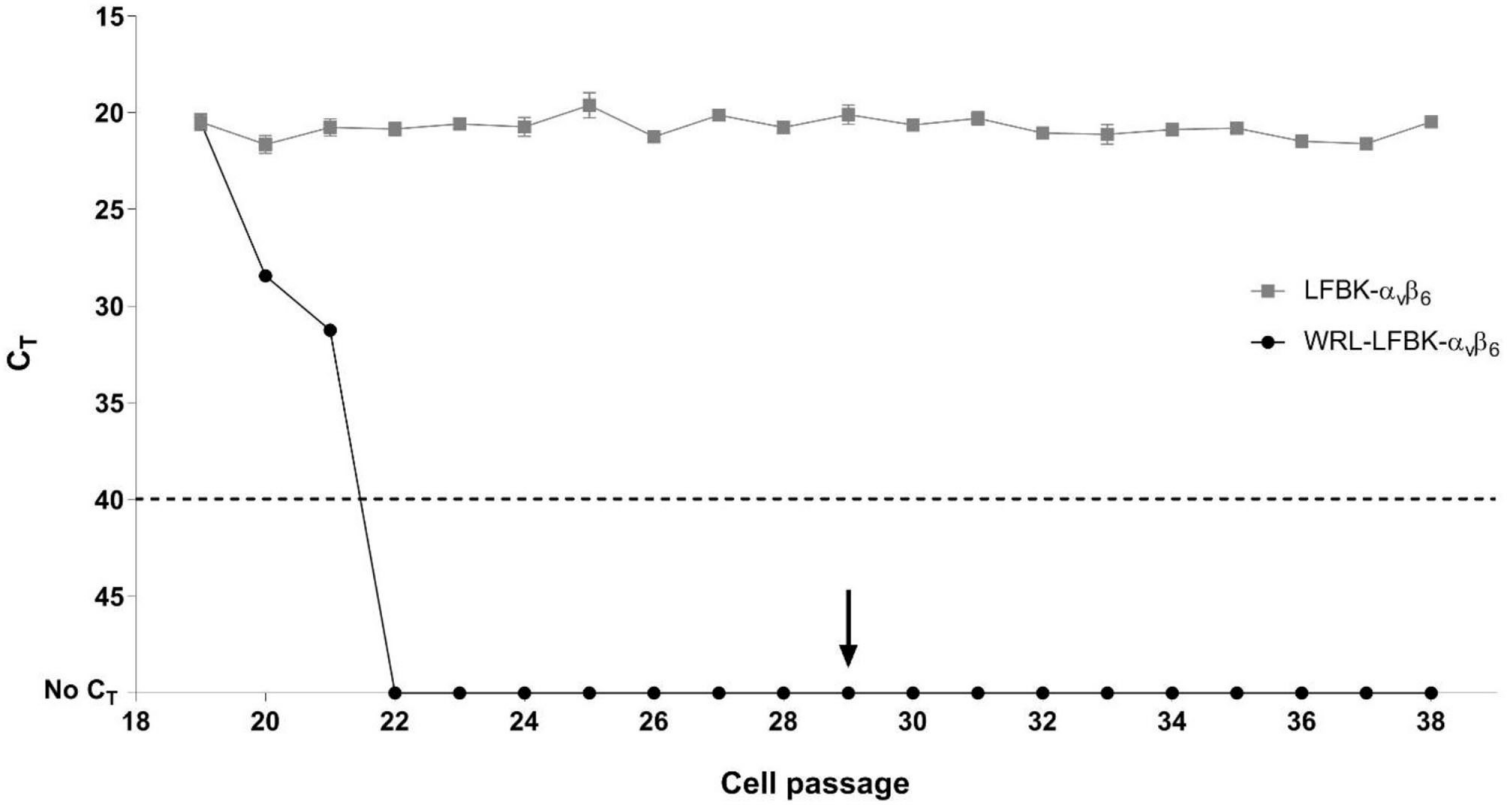
Elimination of BVDV from LFBK-α_v_β_6_ cells as detected by real-time RT-PCR. BVDV was undetected in unclarified cell suspensions of WRL-LFBK-α_v_β_6_ cells after three passages with 4 μM DB772 treatment. Data points represent the average C_T_ (*n* = 3) ± standard deviation. The dotted line denotes the pestivirus assay cut-off (C_T_ of <40), and the arrow indicates the first passage at which DB772 treatment was stopped for the WRL-LFBK-α_v_β_6_ cells.

WRL-LFBK-α_v_β_6_ cell growth was slower during DB772 treatment relative to the untreated controls (10–40% based on estimates of daily confluency), but no cytotoxicity was observed (passages 20–28). BVDV was undetected in WRL-LFBK-α_v_β_6_ cells after three passages in the presence of 4 μM DB772 (Figure 1). The level of BVDV genome in the untreated LFBK-α_v_β_6_ cell line was consistent throughout testing (avg *C*_T_ = 20.8; Figure 1). After treatment with DB772 stopped (starting at passage 29), BVDV remained undetected in WRL-LFBK-α_v_β_6_ cultures through passage 56 (Figure 1, data not shown passages 40–56). The average viral titers were comparable between WRL-LFBK-α_v_β_6_ and LFBK-α_v_β_6_ cell cultures for all vesicular disease viruses tested (*p*-values > 0.05; Table 1).

**Table 1:** Comparative analytical sensitivity of DB772-treated (WRL- LFBK-α_v_β_6_) and untreated cells to vesicular disease viruses.

**Virus**	**Titer (avg Log**_**10**_ **TCID**_**50**_ **/mL** **±** **std dev)**	
	**LFBK-**α_v_β_**6**_	**WRL-LFBK-**α_v6_β_**6**_
FMDV/O/KUW/4/2016 OS	6.7 ± 0.31	6.9 ± 0.31
SVDV/UKG/77/1980 RS1	8.6 ± 0.20	8.5 ± 0.23
SVV MN-88-36695 SK? RS3	9.9 ± 0.46	10.2 ± 0.40
VESV-D53 PK5 RS3	9.1 ± 0.23	9.0 ± 0.35
VSV IND-1 BHK5	7.7 ± 0.31	8.3 ± 0.70

*Values represent the average titer (n = 3) ± standard deviation; there were no significant differences between the two cell lines for each of the viruses tested (p-values > 0.05). OS, original epithelial suspension; cell line abbreviation and passage number: RS, IB-RS-2 cells; BHK, baby hamster kidney cells; PK, pig kidney cells; SK, swine testis cells and ?, unknown passage number*.

## Discussion

In this study, the aromatic cationic compound DB772 was tested at 4 μM to eliminate the non-cytopathic BVDV infection in the LFBK-α_v_β_6_ cell line. After three passages in the presence of DB772, BVDV was undetected and remained undetected throughout subsequent cell passages, even after treatment with DB772 was stopped (Figure 1). The analytical sensitivity of the WRL- LFBK-α_v_β_6_ culture was comparable to the original BVDV-infected LFBK-α_v_β_6_ cell line when titrations of FMDV, SVDV, VSV, VESV, and SVV were tested (Table 1). These data support the use of WRL- LFBK-α_v_β_6_ for FMD and vesicular disease diagnostics, with the findings in agreement to those previously reported in Gray et al. (4).

It is possible that fewer than nine passages with DB772 could have been sufficient to eliminate BVDV from LFBK-α_v_β_6_ cells (i.e., stopping after three passages); however, rRT-PCR testing was not conducted immediately after each passage to confirm the levels of BVDV genome detection. Nonetheless, the presence of DB772 did not appear to have an immediate or long-term effect on the cells (i.e., no cytotoxicity and no difference in viral sensitivity). Cell growth was slower in the presence of DB772, but rebounded after subsequent passaging in DMEM + BS.

In initial studies, Forsythoside A (2-(3,4-Dihydroxyphenyl)ethyl 6-O-(6-deoxy-β-D-gulopyranosyl)-4-O-[(2E)-3-(3,4-dihydroxyphenyl)-2-propenoyl]-α-L-altropyranoside) was also evaluated as a potential compound to eliminate the persistent BVDV infection using an approach similar to that described by Song et al. (21). However, experiments were stopped after the second passage, given that Forsythoside A used at 100 μg/mL in DMEM + BS inhibited LFBK-α_v_β_6_ cell growth.

These results support those of Givens et al. (16), demonstrating that DB772 can be used to eradicate BVDV from persistently infected cells, and highlights the potential use of DB772 for other high value cell lines with non-cytopathic BVDV. Treatment with DB772 and the subsequent elimination of BVDV from LFBK-α_v_β_6_ cells did not alter the ability of these cells to support the growth of FMDV and other vesicular viruses. The BVDV-free cell culture, WRL-LFBK-α_v_β_6_, can be handled using standard precautions associated with cell culture practices, now that the risk of cross-contaminating other cell lines and/or reagents with BVDV is removed. The elimination of BVDV from these cells is also an advantage for preparing challenge strains for *in vivo* studies and vaccine preparations.

## Data Availability Statement

The raw data supporting the conclusions of this article will be made available by the authors, without undue reservation.

## Author Contributions

VM conceived the study. VM and AG developed the methodology. AG and EH conducted the experiments with supervision from BW and VM. AG analyzed these data. BW and VM wrote the manuscript. DK obtained funding. All authors read and approved the manuscript content.

## Conflict of Interest

The authors declare that the research was conducted in the absence of any commercial or financial relationships that could be construed as a potential conflict of interest.

## Publisher's Note

All claims expressed in this article are solely those of the authors and do not necessarily represent those of their affiliated organizations, or those of the publisher, the editors and the reviewers. Any product that may be evaluated in this article, or claim that may be made by its manufacturer, is not guaranteed or endorsed by the publisher.
